# Trends in Hidradenitis Suppurativa Disease Severity and Quality of Life Outcome Measures: Scoping Review

**DOI:** 10.2196/27869

**Published:** 2021-10-01

**Authors:** Jalal Maghfour, Torunn Elise Sivesind, Robert Paul Dellavalle, Cory Dunnick

**Affiliations:** 1 Department of Dermatology Henry Ford Hospital Detroit, MI United States; 2 Department of Dermatology University of Colorado School of Medicine Aurora, CO United States; 3 Dermatology Service Rocky Mountain Regional Veterans Affairs Medical Center Aurora, CO United States

**Keywords:** hidradenitis suppurativa, severity of illness index, patient-reported outcome measures, quality of life, treatment outcome, illness index, patient outcomes, disease severity, Sartorius, dermatology, treatment interventions

## Abstract

**Background:**

Although there has been an increase in the number of randomized controlled trials evaluating treatment efficacy for hidradenitis suppurativa (HS), instrument measurements of disease severity and quality of life (QoL) are varied, making the compilation of data and comparisons between studies a challenge for clinicians.

**Objective:**

We aimed to perform a systematic literature search to examine the recent trends in the use of disease severity and QoL outcome instruments in randomized controlled trials that have been conducted on patients with HS.

**Methods:**

A scoping review was conducted in February 2021. The PubMed, Embase, Web of Science, and Cochrane databases were used to identify all articles published from January 1964 to February 2021. In total, 41 articles were included in this systematic review.

**Results:**

The HS Clinical Response (HiSCR) score (18/41, 44%) was the most commonly used instrument for disease severity, followed by the Sartorius and Modified Sartorius scales (combined: 16/41, 39%). The Dermatology Life Quality Index (18/41, 44%) and visual analogue pain scales (12/41, 29%) were the most commonly used QoL outcome instruments in HS research.

**Conclusions:**

Randomized controlled trials conducted from 2013 onward commonly used the validated HiSCR score, while older studies were more heterogeneous and less likely to use a validated scale. A few (6/18, 33%) QoL measures were validated instruments but were not specific to HS; therefore, they may not be representative of all factors that impact patients with HS.

**Trial Registration:**

National Institute of Health Research PROSPERO CRD42020209582; https://www.crd.york.ac.uk/prospero/display_record.php?ID=CRD42020209582

## Introduction

Hidradenitis suppurativa (HS) is a debilitating chronic inflammatory condition that most commonly involves the axilla, inframammary, inguinal, and anogenital regions [1]. HS is characterized by inflamed nodules that generally progress to painful abscesses, sinus tracts, fibrosis, and scarring [2]. HS has been shown to be associated with the increased incidence of metabolic, autoimmune, and psychosocial comorbidities [2]. Although it has been historically difficult to ascertain the exact prevalence of the disease due to underdiagnosis and variations in the estimates among epidemiologic studies, a recent meta-analysis [3] estimated a worldwide prevalence of 0.3% (range 0.2%-0.6%).

Despite the burden of the disease, the treatment of HS is heterogeneous, and effective management has proven difficult; however, new therapies are under investigation. Randomized controlled trials (RCTs) that are investigating these new therapies have used various instruments to quantify HS disease severity and its impact on patients’ quality of life (QoL).

It is well established that HS results in significant emotional, social, and psychological burdens on patients [4]. Recent studies have reported on the increased prevalence of anxiety, depression, and suicidality among patients with HS [5]. These psychological conditions are indicative of a poor QoL [6] and highlight the importance of incorporating patient-focused outcome measures in HS research. Both the US Federal Drug Administration and European Medicines Agency have recommended the evidence-based use of patient-reported outcome measures (PROMs) in clinical trials and have emphasized their importance [7]. PROMs are particularly important in chronic debilitating skin diseases, such as HS. In HS research, RCTs have reported objective and subjective outcomes via a diverse assortment of scales and questionnaires, making the compilation of data and comparisons between studies quite difficult. A previously published study identified 30 different outcome instruments in HS research [8] and found that nearly 90% of these instruments had not been validated. Given the role of clinical research in providing evidence to inform clinical decision-making, the standardization of outcome measures is crucial to enabling data comparisons between studies.

The purpose of this study was to investigate trends in disease severity scales and QoL instruments that were used in HS-related RCTs conducted between January 1964 and February 2021 via a systematic search of the literature.

## Methods

A scoping review of the literature was conducted in February 2021 by using the following four databases: PubMed, Embase, Web of Science, and Cochrane. To ensure transparency and reproducibility, the literature search was conducted according to the framework established by the PRISMA (Preferred Reporting Items for Systematic Reviews and Meta-Analyses) reporting guidelines [9] and was prospectively registered with PROSPERO. The key search terms were *Hidradenitis Suppurativa*, *acne inversa*, *randomized controlled trial*, *RCT*, *quality of life*, *QoL*, *QOL*, *patient reported outcome measures*, *PROM*, *HS severity*, *severity of HS*, *Sartorius scale*, *Hurley stage*, and *severity of illness index*. Detailed search results are included in Multimedia Appendix 1.

This scoping review included published RCTs that reported disease severity, QoL, or both. Secondary articles (eg, reviews and meta-analyses), case reports and case series, cohort studies, letters to editors, commentaries, and in vivo and in vitro experimental studies were excluded. Two reviewers (JM and TS) independently screened articles to include those that met the defined inclusion criteria, were written in English, and were available as full texts. In total, 111 articles were excluded during title and abstract screening for the following reasons: (1) a non-RCT study design (eg, cohort studies, observational studies, reviews, letters), (2) insufficient data, (3) articles written in languages other than English, and (4) articles that were unavailable in a full-text format. An additional 19 studies were excluded after careful review due to the lack of reporting on disease severity and QoL outcome measures.

## Results

### Summary of Articles

**Figure 1:**
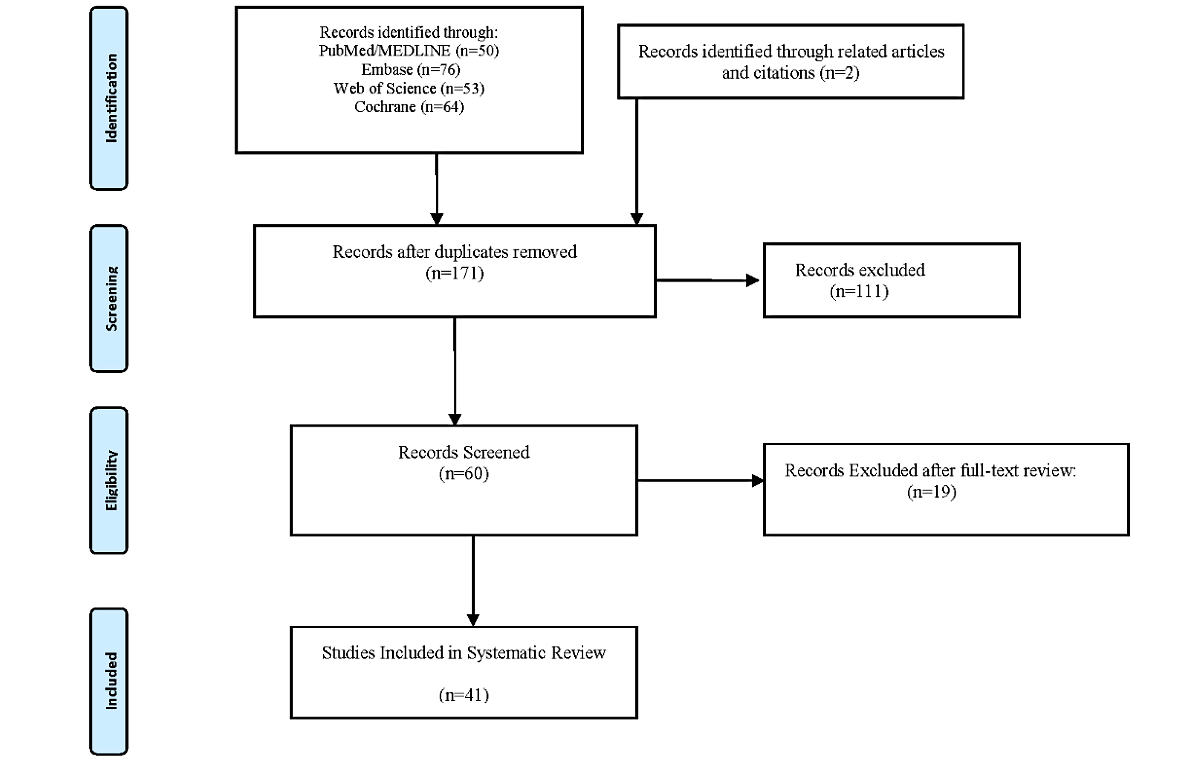
The search process is depicted by using a flow diagram that was adapted from the PRISMA (Preferred Reporting Items for Systematic Reviews and Meta-Analyses) guidelines.

A total of 171 nonduplicated reports were identified; 60 articles underwent a full-text review, and a total of 41 studies [10-50] were included in this review (Figure 1). For each included RCT, the level of evidence was rated according to the evidence levels established by the Oxford Centre for Evidence-Based Medicine [51].

### Data Extraction

The following data were extracted: (1) the proportion of RCTs that used disease severity indices or QoL outcome instruments, (2) the total number of and the frequency of use of disease severity scales, and (3) the total number of and the frequency of use of QoL outcome measures.

### Study Characteristics

A total of 41 RCTs that were published between 1986 and 2021 were identified; these accounted for a total of 3235 participants. The appraisal of studies via the methods outlined by the Oxford Centre for Evidence-Based Medicine evidence ratings scheme was performed; 17 RCTs qualified as level 1b studies, while the remaining 24 studies were level 2b studies. Summary information for the characteristics of the included studies, including evidence levels, is available in Multimedia Appendix 2. Of the 41 included RCTs, 38 (93%) used disease severity outcome measures, and of these 38 RCTs, 30 (79%) used more than 1 scale to assess disease severity. Additionally, 30 of the 41 studies (73%) included QoL measures; of these 30 studies, 20 studies (67%) assessed more than 1 QoL measure.

### Disease Severity Outcome Measures

A total of 25 disease severity outcome measures were identified in this review. The HS Clinical Response (HiSCR) score (19/41, 46%) was the most common instrument used in HS clinical research. HiSCR score use increased from the year 2012 onward. Of the 27 RCTs published since 2012, 18 (67%) used the HiSCR score as an outcome measure (Table 1).

The Sartorius Scale and its modified version—the Modified Sartorius Scale (MSS), which was denoted by some authors as the *Hidradenitis Suppurativa Lesion, Area, Severity Index* (HS-LASI; Sartorius Scale: 7/41, 17%; MSS: 9/41, 22%)—were the second most frequently used instruments for disease severity assessment. The Physician Global Assessment (PGA; 8/41, 20%) was the third most commonly used instrument for disease severity. Only 5 of the 41 RCTs (12%) used the PGA scale that was specific to HS (also known as the HS-PGA). The Hurley stage was primarily used to stratify patients’ disease severity prior to enrollment; only 3 studies incorporated the Hurley stage as an outcome measure. A recently developed and validated HS outcome measure—the International HS Severity Scoring System (IHS4)—was identified in a single RCT that was published in 2021 [50].

There were several studies that used inflammatory markers, including C-reactive protein levels, erythrocyte sedimentation rates, and cytokine profiles (7/41, 17%). Further, 1 RCT used noninvasive sonographic imaging to evaluate lesion depth and vascularity (Table 2).

**Table 1:** The frequency and proportion of disease severity outcome measures.

Disease severity outcome instrument	Studies, n (%)
Hidradenitis Suppurativa Clinical Response^a^	18 (44)
Modified Sartorius Scale^a^; Hidradenitis Suppurativa Lesion, Area, Severity Index; and Modified Hidradenitis Suppurativa Lesion, Area, Severity Index	9 (22)
Physician Global Assessment and evaluation	8 (20)
Sartorius Scale	7 (17)
Hidradenitis Suppurativa Physician Global Assessment^a^	5 (12)
Adverse events	5 (12)
Hurley stage	5 (12)
Abscess and nodule count	5 (12)
Mean improvement in abscesses, fistulae, and nodules	3 (7)
Recurrence	2 (5)
Hidradenitis Suppurativa Severity Index	2 (5)
Time to hidradenitis suppurativa exacerbation	2 (5)
Histological changes	2 (5)
International Hidradenitis Suppurativa Severity Scoring System^a^	1 (2)
Disease Activity Score (visual analogue scale)	1 (2)
Wound healing	1 (2)
Incidence of hidradenitis suppurativa flare	1 (2)
Manchester postinflammatory scar scoring	1 (2)
Hair follicle count	1 (2)
Average number of days to lesion resolution	1 (2)
Investigator and physician assessment	1 (2)

^a^A validated hidradenitis suppurativa scale.

**Table 2:** Laboratory and noninvasive imaging as outcome measures of disease severity.

Diagnostic and inflammatory markers as outcome measures	Studies, n (%)
C-reactive protein	5 (12)
Erythrocyte sedimentation rate	1 (2)
Cytokine profile	1 (2)
Ultrasound findings (eg, vascularity and the depth of lesions)	1 (2)

### QoL Outcome Measures

A total of 18 QoL outcome instruments were identified. These are summarized in Table 3.

The Dermatology Life Quality Index (DLQI) was the most common patient-centered outcome reported in this review (18/41, 44%). A total of 17 studies assessed participants’ pain. Pain was primarily measured by using a visual analogue scale (11/41, 27%) or a numerical ranking (6/41, 15%), although 1 study used the HS-related skin pain scale. In total, 5 of the 41 studies (12%) used the Patient/Participant Global Self-Assessment, which measures various parameters, including pain, pruritus, and disease burden. Patients’ satisfaction with treatment was assessed in 4 of the 41 studies (10%), and 3 RCTs used the Workers Productivity and Impairment Activity Index-Specific Health Problem (WPAI-SHP). Psychological distress was assessed by 2 of the 41 studies (5%), which incorporated the 9-question Patient Health Questionnaire-9 (PHQ-9) depressive symptom scale as a PROM, and by 1 study that used the Hospital Anxiety and Depression Scale (HADS). The European Qol-5 Dimension (EQ-5D), which includes a domain for the assessment of anxiety and depression, was used in 1 RCT.

**Table 3:** Frequency and proportion of quality of life outcome measures.

QoL instrument	Frequency of use in studies, n (%)
Dermatology Life Quality Index^a^	18 (60)
Pain using a visual analogue scale	12 (37)
Pain using a numeric rating scale	6 (20)
Patient/Participant Global Assessment and evaluation	6 (20)
Patient satisfaction	4 (10)
Workers Productivity and Impairment Activity Index-Specific Health Problem^a^	3 (10)
Self-reported pruritus	2 (7)
Patient Health Questionnaire-9^a^	2 (7)
European Quality of Life-5 Dimension^a^	1 (3)
Hidradenitis suppurativa–related skin pain	1 (3)
Patient's overall disease severity and impression (visual analogue scale)	1 (3)
Treatment Satisfaction Questionnaire for Medication^a^	1 (3)
Number of self-reported hidradenitis suppurativa flares	1 (3)
Soreness (visual analogue scale)	1 (3)
Self-assessment of disease burden	1 (3)
Hidradenitis suppurativa–related impairment of general health using a visual analogue scale	1 (3)
Change in the number of daily dressings per week	1 (3)
Hospital Anxiety and Depression Scale^a^	1 (3)

^a^A validated quality of life outcome instrument.

## Discussion

### Principal Findings

HS continues to represent a disease management challenge and result in a substantial disease burden for patients [2]. Our review of 41 RCTs (published in English) identified 25 disease severity measurements (Tables 1 and 2) and 18 QoL instrument scales (Table 3). Overall, we identified a diverse assortment of outcome measures, which may indicate a barrier to their synthesis and translation into clinical practice.

With regard to the validity of the outcome measures identified in our review, both the HS-PGA and HiSCR score have been shown to be valid assessments, with HiSCR being the most extensively validated outcome measure in published RCTs. The two most commonly reported disease severity scales in our study—the HiSCR score and the Sartorius Scale and MSS—differ substantially in their approaches and frames of reference; the HiSCR assesses clinical responses from baseline (namely, a reduction in inflammatory lesion count), and the Sartorius Scale and MSS assess the extent of HS inflammation by counting anatomic regions and the types and numbers of lesions.

The HiSCR score was the only validated scoring system that appeared in the studies reviewed (all reviewed articles: 18/41, 44%; articles reporting disease severity as an outcome: 18/38, 47%), making it the most commonly used validated scale in HS research. The HiSCR score is a validated scoring system that is used to evaluate treatment response, and it has been shown to be reliable in both clinical research and daily practice [24]. Although the minimal clinically important difference for HiSCR scores has not been established, a 50% reduction in the total abscess and nodule count appears to be meaningful to both patients and physicians [33].

Although only 5 RCTs used the HS-PGA, it is important to highlight that it is considered to be a relatively easy scoring system that assesses treatment efficacy in clinical research. Similar to the HiSCR score, it is a dynamic outcome instrument that can be used to monitor disease progression [52]. However, compared to the HiSCR score, the HS-PGA has a lower sensitivity for rapidly identifying changes in HS-specific lesions. For instance, some patients with severe HS-specific lesions can experience clinically important improvements without achieving meaningful reductions in their HS-PGA scores [52].

The Sartorius Scale, which is widely used to assess clinical responses to treatment based on the involved anatomical regions and the number and type of lesions involved (nodules, fistulae, and abscesses), the distance between lesions, and whether normal skin exists between lesions, poses a challenge to results interpretation [53]. In addition to being only partially validated, the Sartorius Scale may be quite time consuming to administer and difficult to replicate in a busy outpatient clinic.

The MSS (or HS-LASI) represents a more streamlined version of the original Sartorius Scale; the MSS includes a reduced number of specific types of lesions and a reduced number of points for each parameter [54]. Although it is simpler than the traditional Sartorius Scale, the MSS (or HS-LASI) remains time-consuming and difficult to interpret in the context of assessing coalescing and large lesions. In this review, we identified 2 RCTs that used the HS-LASI scale [22,27], whereas 11 RCTs used a combination of both the MSS and the traditional Sartorius Scale. The overlap in the naming and content of the Sartorius Scale and its variants, such as the MSS and HS-LASI, can hinder meaningful comparisons between studies and thus create challenges in interpreting data and making informed clinical decisions.

In 2016, Ingram et al [8] found that 90% of outcome measures that are used in HS research are not validated; however, the research landscape appears to be changing. We found that RCTs published from 2014 onward were more likely to use the validated HiSCR scale, while older studies used more diverse outcome measures, of which many had low interobserver reliability [55], and were less likely to have used a validated scale. In 2018, the HS ALLIANCE working group highlighted the need to incorporate validated outcome measures and PROMs in HS research [56]. In 2017, the members of the European HS Foundation demonstrated the validity of a novel instrument—the IHS4 [57]. The IHS4 has been shown to be a dynamic instrument for assessing HS severity and is applicable to both clinical research and daily clinical practice [57]. We found a single, recent RCT (published in 2021) that used the IHS4 as an outcome measure [50].

As with instruments of disease severity assessment, patient-reported QoL measures demonstrate significant heterogeneity and are generally nonspecific [57]. Although the majority of articles (30/41, 73%) discussed the impact of HS on patients’ lives, the instruments that were used remain inadequate for capturing the overall impact of disease burden on patients. Of all of the QoL instruments identified in this review, the DLQI appeared in 44% (18/41) of RCTs, making it the most commonly used patient-centered instrument in HS research. The DLQI is a validated instrument that is widely used for an array of dermatologic conditions, such as psoriasis and atopic dermatitis, but is not specific to HS.

In addition to QoL instruments, specific outcomes pertaining to pain assessment are needed. Although the visual analogue pain scale has been validated in clinical research, it is not specific to HS. Despite various treatment options, a recent survey study revealed that inadequate pain management is perceived as an unmet need by both patients and health care providers [58]. Given that pain is associated with psychosocial comorbidities [34], it is essential to develop specific core outcome scales that assess pain management and treatment responses.

In contrast to disease severity outcome measures, we identified 6 validated QoL instruments. These include the DLQI, PHQ-9, HADS, EQ-5D, WPAI-SHP, and Treatment Satisfaction Questionnaire for Medication [59]. However, these are not HS-specific QoL instruments. The emotional, social, and psychological impacts of HS on patients cannot be overstated; while QoL can be measured in various ways, the current QoL instruments that are used in HS research may not adequately capture changes that specifically pertain to the HS population.

In 2018, the first HISTORIC (HS Core Outcomes Set International Collaboration) Delphi study [60] reached a consensus on the following five core domains that are relevant to all types of clinical research: pain, physical signs, HS-specific QoL, global assessment, and the progression of the disease course. HISTORIC Delphi also developed the HS QoL (HiSQOL) scale—an HS-specific QoL instrument [61].

Over the past several years, there has been an increased effort to develop validated, HS-specific QoL outcome instruments, including the aforementioned HiSQOL scale, the HIDRAdisk, and the 44-item HS-QoL questionnaire [61-64]. Promising HS-specific QoL instruments such as these may soon be incorporated in future clinical trial outcome measurements.

Kimball et al [65] introduced the following two specific questionnaires in 2018: the HS Symptom Assessment (HSSA) and the HS Impact Assessment (HSIA). Both the HSSA and HSIA are validated instruments and are considered to be reliable tools for assessing symptoms and the efficacy of HS treatment. We identified no RCTs that used these two instruments for the evaluation of therapeutic interventions for HS.

Ongoing research may soon allow for new technologies to supplement the clinical assessment of HS lesion severity, which relies, in part, on manual palpation–noninvasive imaging techniques such as medial infrared thermography, and may soon aid in the evaluation of disease state and treatment response [66]. The broader adoption of standardized, validated QoL and disease severity measurement tools may allow for the better assessment of the overall impact of disease burden on patients, including the effect of HS on mental health [65], which, in our review, was not well characterized by the limited patient outcome measures reported.

### Limitations

The limitations of this review include that it was restricted to published RCTs and that it excluded other types of publications, such as cohort studies, case control studies and case series, and ongoing or current clinical trials, that may provide further insight. We chose to include RCTs exclusively, as it was a priority to assess evidence of the highest level. It is unclear if other studies with less rigorous methods have similar trends in reporting disease severity and QoL outcome measures—an area that remains open for further follow-up studies. None of the included studies in this review involved pediatric participants; therefore, the trends in outcome measures that we identified may not be applicable to this population group. In addition, this review did not explore the utility of HS interventions and therefore cannot add to the body of knowledge regarding treatment efficacy in HS.

### Conclusion

This review highlights the heterogeneity of outcome measures that are used in RCTs to assess disease severity and QoL for patients with HS. Among the 41 English RCTs published from 1964 to 2021, the HiSCR score remained the predominant outcome instrument that was used to assess HS disease severity. The IHS4, which is representative of an expanding number of validated disease severity outcome measures, was used in only 1 RCT among those published from 1964 to 2021. Patient QoL measures remain central to evaluating disease impact and the degree of improvement for patients in clinical studies. PROMs are gaining importance in clinical research and are strongly supported by guidance from both the US Federal Drug Administration and European Medicines Agency. Recently developed instruments with proven validity, such as the HSSA, HSIA, and HiSQOL scale, represent advancements in measuring the QoL outcomes of HS. Our findings underscore the need for standardized outcome measures that are essential for comparability among studies and the improved quality of research evidence.

## Appendix

Multimedia Appendix 1 Search strategies.

Multimedia Appendix 2 Study characteristics and evidence levels.

## Abbreviations

DLQI: Dermatology Life Quality Index
EQ-5D: European Quality of Life-5 Dimension
HADS: Hospital Anxiety and Depression Scale
HiSCR: Hidradenitis Suppurativa Clinical Response
HiSQOL: Hidradenitis Suppurativa Quality Of Life
HISTORIC: Hidradenitis Suppurativa Core Outcomes Set International Collaboration
HS: hidradenitis suppurativa
HSIA: Hidradenitis Suppurativa Impact Assessment
HS-LASI: Hidradenitis Suppurativa Lesion, Area, Severity Index
HSSA: Hidradenitis Suppurativa Symptom Assessment
IHS4: International Hidradenitis Suppurativa Severity Scoring System
MSS: Modified Sartorius Scale
PGA: Physician Global Assessment
PHQ-9: Patient Health Questionnaire-9
PRISMA: Preferred Reporting Items for Systematic Reviews and Meta-Analyses
PROM: patient-reported outcome measure
QoL: quality of life
RCT: randomized controlled trial
WPAI-SHP: Workers Productivity and Impairment Activity Index-Specific Health Problem
